# PET-based radiomic feature based on the cross-combination method for predicting the mid-term efficacy and prognosis in high-risk diffuse large B-cell lymphoma patients

**DOI:** 10.3389/fonc.2024.1394450

**Published:** 2024-06-05

**Authors:** Man Chen, Jian Rong, Jincheng Zhao, Yue Teng, Chong Jiang, Jianxin Chen, Jingyan Xu

**Affiliations:** ^1^ Department of Hematology, Nanjing Drum Tower Hospital, School of Basic Medicine and Clinical Pharmacy, China Pharmaceutical University, Nanjing, China; ^2^ The Key Laboratory of Broadband Wireless Communication and Sensor Network Technology (Ministry of Education), Nanjing University of Posts and Telecommunications, Nanjing, China; ^3^ Department of Nuclear Medicine, Nanjing Drum Tower Hospital, Affiliated Hospital of Medical School, Nanjing University, Nanjing, China; ^4^ Department of Nuclear Medicine, West China Hospital, Sichuan University, Chengdu, China; ^5^ Department of Hematology, Nanjing Drum Tower Hospital, Affiliated Hospital of Medical School, Nanjing University, Nanjing, China

**Keywords:** [18F]-FDG PET/CT, diffuse large B-cell lymphoma, machine learning, interim, treatment outcome, prognosis

## Abstract

**Objectives:**

This study aims to develop 7×7 machine-learning cross-combinatorial methods for selecting and classifying radiomic features used to construct Radiomics Score (RadScore) of predicting the mid-term efficacy and prognosis in high-risk patients with diffuse large B-cell lymphoma (DLBCL).

**Methods:**

Retrospectively, we recruited 177 high-risk DLBCL patients from two medical centers between October 2012 and September 2022 and randomly divided them into a training cohort (n=123) and a validation cohort (n=54). We finally extracted 110 radiomic features along with SUVmax, MTV, and TLG from the baseline PET. The 49 features selection-classification pairs were used to obtain the optimal LASSO-LASSO model with 11 key radiomic features for RadScore. Logistic regression was employed to identify independent RadScore, clinical and PET factors. These models were evaluated using receiver operating characteristic (ROC) curves and calibration curves. Decision curve analysis (DCA) was conducted to assess the predictive power of the models. The prognostic power of RadScore was assessed using cox regression (COX) and Kaplan–Meier plots (KM).

**Results:**

177 patients (mean age, 63 ± 13 years,129 men) were evaluated. Multivariate analyses showed that gender (OR,2.760; 95%CI:1.196,6.368); *p*=0.017), B symptoms (OR,4.065; 95%CI:1.837,8.955; *p*=0.001), SUVmax (OR,2.619; 95%CI:1.107,6.194; *p*=0.028), and RadScore (OR,7.167; 95%CI:2.815,18.248; *p*<0.001) independently contributed to the risk factors for predicting mid-term outcome. The AUC values of the combined models in the training and validation groups were 0.846 and 0.724 respectively, outperformed the clinical model (0.714;0.556), PET based model (0.664; 0.589), NCCN-IPI model (0.523;0.406) and IPI model (0.510;0.412) in predicting mid-term treatment outcome. DCA showed that the combined model incorporating RadScore, clinical risk factors, and PET metabolic metrics has optimal net clinical benefit. COX indicated that the high RadScore group had worse prognosis and survival in progression-free survival (PFS) (HR, 2.1737,95%CI: 1.2983, 3.6392) and overall survival (OS) (HR,2.1356,95%CI: 1.2561, 3.6309) compared to the low RadScore group. KM survival analysis also showed the same prognosis prediction as Cox results.

**Conclusion:**

The combined model incorporating RadScore, sex, B symptoms and SUVmax demonstrates a significant enhancement in predicting medium-term efficacy and prognosis in high-risk DLBCL patients. RadScore using 7×7 machine learning cross-combinatorial methods for selection and classification holds promise as a potential method for evaluating medium-term treatment outcome and prognosis in high-risk DLBCL patients.

## Introduction

Diffuse large B-cell lymphoma (DLBCL) is a highly heterogeneous and aggressive B-cell lymphoma, accounting for 30%-40% of initial diagnosed non-Hodgkin’s lymphomas (NHL) (1). The first-line immunochemotherapy are R-CHOP (rituximab, cyclophosphamide, doxorubicin, vincristine and prednisone) or R-CHOP-like regimens (2, 3). Clinically, 30%-40% of patients undergoing this therapy experience relapse or refractory (4, 5). This could be attributed to the tumor heterogeneity, leading to reduced sensitivity to chemotherapy (6, 7). Patients classified as high-risk face poorer prognostic survival (8). The gene expression profiling of DLBCL defined three primary subtypes based on “cell of origin” (COO): germinal center B cell-like (GCB), activated B cell-like (ABC), and not otherwise specified (NOS). The molecular subclassification could account for some of the heterogeneity in the clinical outcomes of DLBCL (9). Numerous prognostic tools have been identified through large-scale retrospective studies. The International Prognostic Index (IPI) was proposed in 1993, incorporating five risk factors: age, lactate dehydrogenase (LDH), the Eastern Cooperative Oncology Group (ECOG) Physical Status (PS), Ann Arbor stage, and extra-nodal involvement (10). The National Comprehensive Cancer Network -IPI (NCCN-IPI) was proposed in 2014, which form four risk groups based on scores ranging from 0 to 8. The NCCN-IPI provides more accurate identification of intermediate-high (4, 5) /high-risk (6–8) DLBCL patients (11). However, the focus of both the IPI and the NCCN-IPI on clinical and biologic indicators makes it difficult to comprehensively assess the tumor heterogeneity of DLBCL (12, 13).

18F-fluorodeoxyglucose (FDG)-positron emission tomography/computed tomography (PET/CT) is widely utilized for early DLBCL diagnosis, staging, and assessing chemotherapy response (14). SUVmax, MTV and TLG are commonly used in PET. These metabolic indicators reflect tumor malignancy and are valuable for baseline assessment as well as improve response prediction (15). In the previous research, SUVmax is the most widely used indices (16). MTV and TLG, are associated with tumor burden, as well as progression-free survival (PFS) and overall survival (OS) (17). Vercellino et al. found that the integration of baseline total metabolic tumor volume (TMTV) with parameters of tumor load distribution has the potential to enhance the accuracy of risk stratification for DLBCL patients (18). Nevertheless, these indicators have limitations on describing tumor heterogeneity. Radiomics were used to assess tumor heterogeneity and assisted in the prediction of clinical outcomes. PET radiomics features present promising biomarkers for predicting treatment outcome and prognosis in DLBCL (19).

Machine learning is commonly used for radiomic feature identification and classification (20). Several studies investigated the risk stratification and efficacy of PET radiomics, Lue et al. used the least absolute shrinkage and selection operator regression (LASSO) method and discovered that the baseline 18F-FDG PET radiomic feature RLN_GLRLM_ is an independent prognostic factor for survival outcomes (21). But these studies utilized limited machine learning methods (22, 23). Additionally, other studies reported the outcome and prognostic value of radiomics features using cross-combination methods (24). However, these methods have not yet been applied in high-risk DLBCL patients. In this paper, we therefore employed a cross-combination of seven machine learning methods to select and classify PET radiomics features associated with tumor internal heterogeneity. Furthermore, we established a tool as early prognostic biomarker that predicts mid-term treatment outcome and prognosis, also identifies high-risk DLBCL patients with unresponsive to R-CHOP regimen.

## Materials and methods

### Patient data collection

This study followed the principles outlined in the Declaration of Helsinki. Ethical approval for this retrospective analysis was obtained from the Ethics Committee of two medical centers. Written consent was not required for this study. A total of 177 patients with DLBCL classified as intermediate-high/high-risk according to NCCN-IPI score of 4–8 were enrolled between October 2012 and September 2022. Among them, 125 patients were from Nanjing Drum Tower Hospital of Nanjing University Medical School, and 52 patients were from West China Hospital of Sichuan University. The patients were randomly divided into a training cohort (123) and a validation cohort (54) using a 7:3 randomization ratio. Inclusion criteria were defined as follows: (I) patients with confirmed NCCN-IPI ≥4 for DLBCL, (II) [18F]-FDG PET/CT scan was performed before baseline treatment, and (III) received R-CHOP-like regimens, and (IV) patients had to be aged ≥18 years at the time of diagnosis. Exclusion criteria were used: (I) participants with primary central nervous system lymphoma, (II) participants with a history of other tumors, and (III) participants with incomplete clinical data, and (IV) had undergone previous treatment such as chemotherapy, radiotherapy, or surgery, and (V) lost to follow-up.

**Figure 1:**
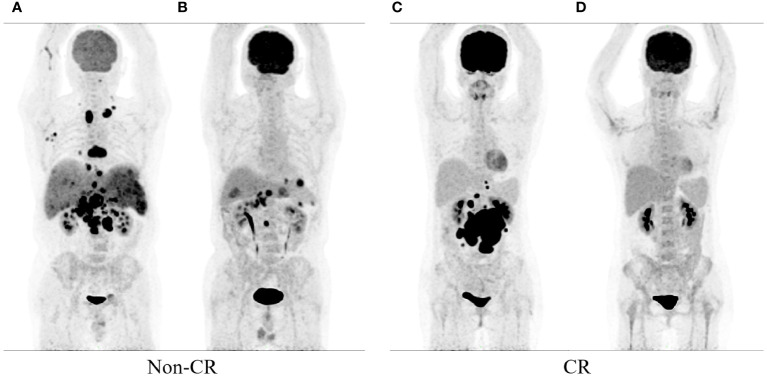
show the baseline and mid-term 18F-FDG PET/CT of the patients. Baseline **(A)** and mid-term image **(B)** of the patient without complete remission (Non-CR), and baseline **(C)** and mid-term image **(D)** of the patient with complete response (CR).

The datasets included patient clinical data such as gender, age, B symptoms, ECOG PS, IPI, NCCN-IPI, LDH, Ann Arbor stage, extranodal involvement, bone marrow involvement. Patient follow-up data were collected through electronic medical records or telephone interviews. The mid-term PET scans based on the Deauville 5-point scale were used as study endpoints for mid-term efficacy and prognosis in DLBCL patients. A score of 1–3 was defined as complete metabolic remission (CMR), and 4–5 was defined as partial metabolic remission (PMR), disease stabilization (SD), or disease progression (PD) (25). Therefore, the patients were divided into CR group and non-CR group. **Figure 1** illustrates the baseline and mid-term pet of non-CR and CR patients.

### PET/CT scanning protocol

All patients should fast for more than 6 hours before PET/CT scans, and their fasting blood glucose levels were under 8.7 mmol/L. Patients were injected with 18F-FDG (3.70–5.18 MBq/kg; Fludeoxyglucose[18F] Injection; AMS Limited) via a superficial forearm vein, and rested quietly for 60 minutes before PET/CT. CT scanning conditions included a tube voltage of 120 kV, tube current of 100 mA, and layer thickness of 2 mm (Philips). PET scanning conditions included acquisition of 7–10 beds, with each bed lasting for 2 minutes (Philips2). At the end of acquisition, a response line image reconstruction was implemented to obtain cross-sectional, coronal, and sagittal PET and CT images, which were later corrected for attenuation. Image reconstruction was performed using voxels of 4 × 4 × 4 mm³ over three iterations and 33 subsets.

### VOI drawing and radiomics processing

The PET images were processed using LIFEx (Local Image Feature Extraction) software(version7.3.0) (26). (I) A voxel boundary threshold of 41% SUVmax was employed (15). A semi-automatic segmentation method was used to outline the volume of interest (VOI), (II) with non-lymphoma 18F-FDG uptake being manually excluded. In case of disagreement, a senior nuclear medicine physician was consulted to jointly determine the VOI. (III) The metabolic metrics, including SUVmax, MTV, and TLG, were determined for each lesion. SUVmax represented the maximum standardized uptake value with the highest uptake in tumor lesions. MTV was the volume of tumor lesion for a single VOI, and TLG was calculated as the sum of the product of the SUVmean and the MTV for the lesion (TLG = [SUVmean × MTV]). Lesions with MTV smaller than 10 cm³ were not included. All radiomics features complied with the benchmarks of the Image Biomarker Standardization Initiative (IBSI) (27).

**Figure 2:**
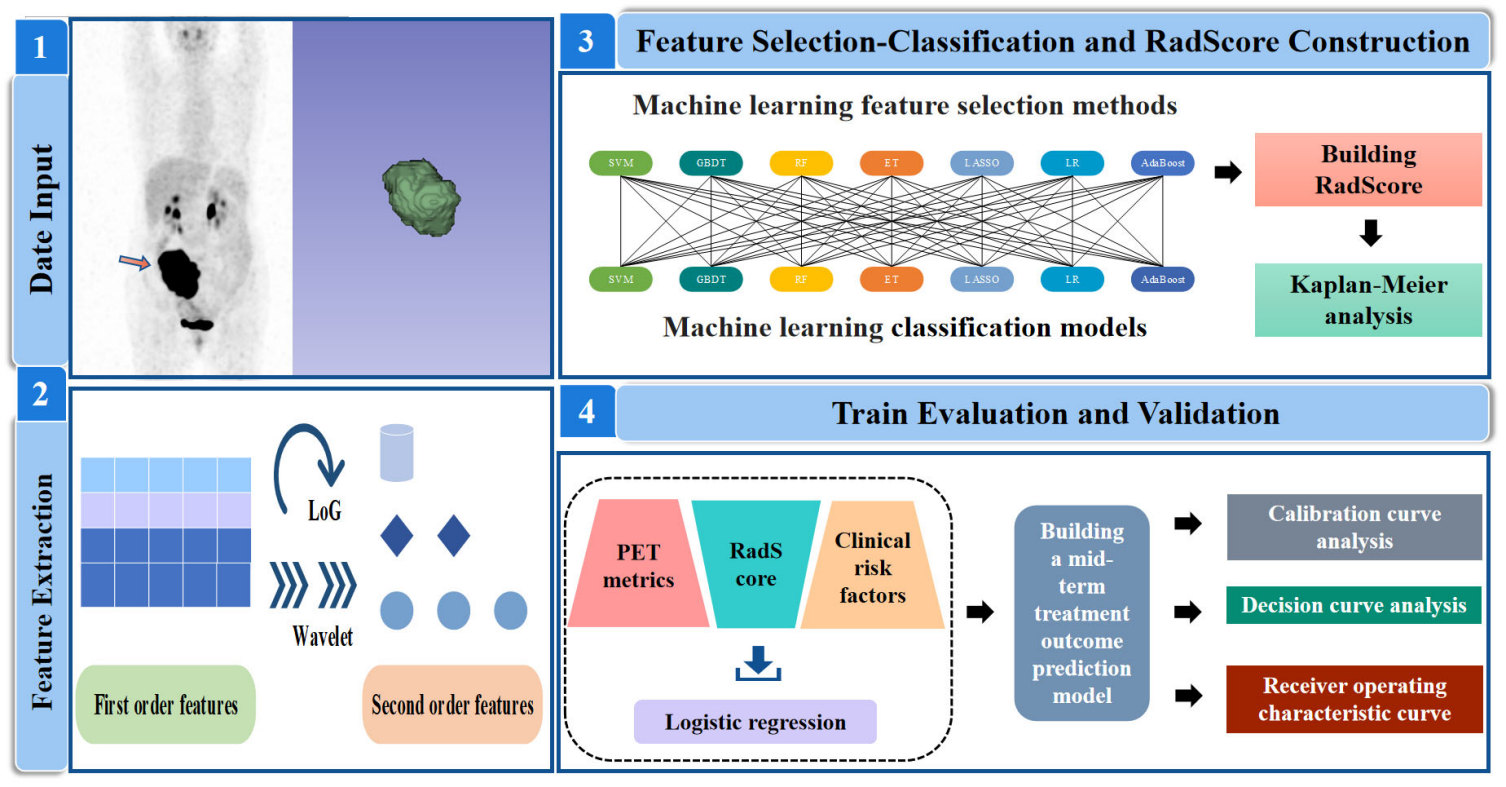
Analysis workflow in this study. SVM, support vector machine; GBDT, gradient boosting decision tree; RF, random forest; ET, extra-trees; LASSO, least absolute shrinkage and selection operator; LR, logistic regression; AdaBoost, adaptive boosting.

PET radiomic features were extracted from baseline PET images by the open-source software package LIFEx (www.lifexsoft.org). For the original PET image, (I) the Wavelet and Laplacian of Gaussian (LoG) transform were applied to obtain the corresponding Wavelet and LoG images. Then, (II) three types of features were extracted: first-order statistical features (maximum, minimum), shape features (roundness, extensibility), and texture features. **Figure 2** illustrates the workflow of radiomic analysis.

### Radiomics feature selection and RadScore construction

The extracted radiomic features were screened and classified using a cross-combination method of seven machine learning models. These methods are Gradient Boosted Decision Tree (GBDT), Extreme Tree (ET), Random Forest (RF), Adaptive Boosting (AdaBoost), Least Absolute Shrinkage and Selection Operator (LASSO), Support Vector Machines (SVM) and Logistic Regression (LR). GBDT (28) utilizes decision trees as its base learner, with predictions from a series of trees summed together. RF (29) is an ensemble of decision trees, where the results of all the decision trees are voted upon or averaged to obtain the final prediction. ET (30) is the model underlying the feature recursive elimination algorithm, which selects the dataset and obtains weight values for each feature. Features with the smallest absolute weight values are then sequentially removed from the feature set. AdaBoost (31) adapts to different datasets by adjusting the weights of the training samples and combines multiple classifiers linearly to enhance their performance. LASSO (32) is a classical regression analysis method that minimizes regression coefficients through shrinkage operations, preserving non-zero variables in the model. SVM (33) is a powerful method for building classifiers that establishes a decision boundary between two categories, enabling label prediction based feature vectors. LR (34) is a generalized linear model used for classification tasks, analyzing the impact of independent variables on classification results by quantifying their effects.

This paper presented a feature selection-classification pairs from 7×7 possible combinations, such as LASSO-LASSO SVM-SVM and SVM-LASSO. Seven machine learning methods were used to select features, and seven machine learning methods were used to classify features. Subsequently, the optimal candidate pair were used to build Radiomic Score (RadScore). RadScore was defined as the sum of the product of the selected radiomic feature and the corresponding feature weights. The identification of the best candidate model involved five steps utilizing fivefold cross-validation: (I) The patient data was randomly divided into training(n=123) and validation(n=54) cohorts. (II) For the training cohort, we employed seven feature selection models, developed 110 PET radiomics features and obtained corresponding feature weights after dimensionality reduction. Based on these feature weights, we trained feature selection models by recursively considering subsets of radiomic features. The feature selection model with the largest area under curve (AUC) value was identified as the most important one. Then(III) fivefold cross-validation was applied to the reduced training cohort that divided it into approximately equal-sized groups, with four groups used for training and one group for test. (IV) Four training groups had been separately developed using the seven feature classification models. The feature classification model with the largest AUC value was identified as the most important one. (V) We calculated the AUC of each feature selection-classification model and outputted the average AUC. The model with the largest average AUC was selected as the optimal candidate model. (VI) Finally, we validated the optimal model in the test group.

### Development and validation of the models

Univariate and multivariate logistic regression were utilized to identify potential independent risk factors in the training group and construct a predictive model for the mid-term treatment outcome. In the univariate analysis, statistically significant clinical and PET factors were included separately in the multivariate analysis. Independent clinical predictors were employed to develop clinical models, while independent PET predictors were utilized to create PET-based models. Subsequently, all independent clinical predictors, PET predictors, and RadScore were assembled a combined model. Additionally, NCCN-IPI model and IPI model were also developed.

### Clinical benefit analysis based on the models

All models were assessed in both the training and validation groups through Receiver operating characteristic (ROC) curves and calibration curves. Additionally, decision curve analysis (DCA) was employed to evaluate the net clinical benefits of these models.

### Statistical analysis

All data were analyzed using SPSS 25.0 (IBM Corp, Armonk, NY, USA) and R statistical software (version 4.2.2). A *P* value less than 0.05 was considered statistically significant. The χ2 test was used to compare clinical characteristics and PET metabolic metrics in the training and validation groups. Nomograph were used to show the score of independent risk factors. ROCcurves were utilized to determine the optimal thresholds for SUVmax, MTV, TLG, and RadScore in predicting mid-term efficacy, PFS and OS. Logistic regression analyses were employed to assess and develop independent predictors. Calibration curves, ROC, and DCA were calculated for the model in both the training and validation cohorts. Survival analysis was conducted by Cox regression and Kaplan-Meier (KM) analysis.

## Results

### Patient characteristics

A total of 177 patients (mean age,63 ± 13 years,129 men) were included. **Table 1** summarized the baseline characteristics for patients in both the training and validation cohorts. The χ2 test revealed no statistically significant(*P*<0.05) difference between the two groups. The median follow-up time for the training and validation cohorts was 30.5 and 30.8 months, respectively. In the training cohort, 62 individuals experienced disease relapse or progression, resulting in 42 deaths. The 1-year, 3-year, and 5-year PFS rates were 89.6%, 72.2%, and 56.2%, while 1-year, 3-year, and 5-year OS rates were 89.3%, 67.9%, and 63.4%. Likewise, in the validation cohort, disease relapse or progression occurred in 24 individuals, leading to 13 deaths. 1-year, 3-year, and 5-year PFS rates were 79.7%, 55.3%, and 38.0%, and 1-year, 3-year, and 5-year OS rates were 90.9%, 75.8%, and 70.0%.

**Table 1:** Demographics and clinical characteristics of the study population.

Category	Characteristic	Training cohort (*n=123*)	Validation cohort (*n=54*)	*P* value
Clinical predictors	Gender Female Male	29 (23.6%) 94 (76.4%)	19 (35.2%) 35 (64.8%)	0.110
	Age (year) <60 ≥60	52 (42.3%) 71 (57.7%)	29 (53.7%) 25 (46.3%)	0.132
	LDH level normal elevate(≥245U/L)	102 (82.9%) 21 (17.1%)	40 (74.1%) 14 (25.9%)	0.173
	B symptoms			
PET predictors	no yes ECOG PS 0–1 ≥2 IPI 0–2 ≥3 NCCN-IPI 4–5 6–8 Ann Abor stage I-II III-VI Extranodal involvement 0–1 ≥2 Bone marrow involvement no yes Bulky disease no yes Pathological type GCB Non-GCB SUVmax <19.2 ≥19.2 MTV (cm^3^) <25.6 ≥25.6 TLG <222.0 ≥222.0	86 (69.9%) 37 (30.1%) 20 (16.3%) 103 (83.7%) 100 (81.3%) 23 (18.7%) 106(86.2%) 17(13.8%) 13 (10.6%) 110 (89.4%) 91 (74.0%) 32 (26.0%) 29 (23.6%) 94 (76.4%) 77 (62.6%) 46 (37.4%) 71 (57.7%) 52 (42.3%) 74 (61.0%) 49 (39.0%) 33 (26.8%) 90 (73.2%) 43 (35.0%) 80 (65.0%)	33 (61.1%) 21 (38.9%) 12 (22.2%) 42 (77.8%) 40 (74.1%) 14 (25.9%) 46(85.2%) 8(14.8%) 6 (11.1%) 48 (88.9%) 39 (72.2%) 15 (27.8%) 20 (37.0%) 34 (63.0%) 33 (61.1%) 21 (38.9%) 29 (53.7%) 25 (46.3%) 24 (44.4%) 30 (55.6%) 16 (29.6%) 38 (70.4%) 19 (35.2%) 35 (64.8%)	0.250 0.343 0.276 0.861 0.915 0.807 0.065 0.851 0.619 0.053 0.701 0.977
Radiomics predictors	RadScore <2.0 ≥2.0	54 (43.9%) 69 (56.1%)	24 (44.4%) 30 (55.6%)	0.947

F, female; M, male; LDH, lactate dehydrogenase; ECOG PS, Eastern Cooperative Oncology Group performance status; IPI, International Prognostic Index; NCCN-IPI, National Comprehensive Cancer Network International Prognostic Index; GCB, germinal centre B cell; SUVmax, maximum standardized uptake value; MTV, metabolic tumor volume; TLG, total lesion glycolysis; RadScore, Radiomics Score.P value was derived from the χ2 test.

### Radiomics feature selection and RadScore construction

**Figure 3:**
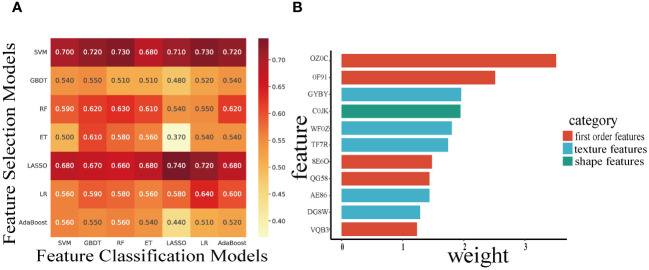
Heatmaps indicate the AUC performance of the cross-combinations of the feature selection methods (columns) and classification models (rows) in predicting mid-term response **(A)**. The Histogram demonstrate the selected features (IBSI name) and weights to build the optimal candidate model **(B)**.

Based on the 49 features machine learning selection-classification pairs, we selected 110 radiomics features to construct the optimal LASSO-LASSO model (AUC=0.74) (**Figure 3**). The LASSO-LASSO model screened out 10 key radiomics features for constructing RadScore (**Table 2**). We employed the ROC curves to identify the optimal cut-off for these dichotomous variables, which corresponds to the point with the maximum Youden index. The Youden index represents the sum of sensitivity and specificity and then subtracting 1. **Table 3** shows that RadScore cut-off threshold of 2.0, 2.2 and 2.2 was optimal for predicting mid-term efficacy, PFS and OS.

**Table 2:** The 110 radiomic features extracted from PET and the 11 key features* for constructing RadScore in this study.

Classification	Radiomics feature	IBSI name
shape feature	Volume; Approximate Volume; Surface Area*; Surface To Volume Ratio; Compactness1; Compactness2; Spherical Disproportion; Sphericity; Asphericity; Centre Of Mass Shift; Maximum 3D; Diameter; Integrated Intensity	RNU0; YEKZ; C0JK*; 2PR5; SKGS; BQWJ; KRCK; QCFX; 25C7; KLMA; L0JK; 99N0
first-order feature	Mean; Variance; Skewness; Kurtosis; Median; Minimum Grey Level; 10th Percentile*; 50th Percentile; 90th Percentile; Maximum Grey Level; Inter quartile Range; Range; Mean Absolute Deviation; Robust Mean Absolute Deviation; Median Absolute Deviation; Coefficient Of Variation; Quartile Coefficient Of Dispersion; Energy; Root Mean Square; Global Intensity Peak*; Intensity Histogram Mean; Intensity Histogram Variance; Intensity Histogram Skewness; Intensity Histogram Kurtosis; Intensity Histogram Median; Intensity Histogram Minimum Grey Level; Intensity Histogram 10th Percentile; Intensity Histogram 90th Percentile*; Intensity Histogram Maximum Grey Level; Intensity Histogram Mode; Intensity Histogram Inter quartile Range; Intensity Histogram Range; Intensity Histogram Mean Absolute Deviation; Intensity Histogram Robust Mean Absolute Deviation; Intensity Histogram Median Absolute Deviation; Intensity Histogram Coefficient Of Variation; Intensity Histogram Quartile Coefficient Of Dispersion; Intensity Histogram EntropyLog2; Uniformity; Maximum Histogram Gradient; Maximum Histogram Gradient Grey Level*; Minimum Histogram Gradient*; Minimum Histogram Gradient Grey Level	Q4LE; ECT3; KE2A; IPH6; Y12H; 1GSF; QG58*; Y12H.1; 8DWT; 84IY; SALO; 2OJQ; 4FUA; 1128; N72L; 7TET; 9S40; N8CA; 5ZWQ; 0F91*; X6K6; CH89; 88K1; C3I7; WIFQ; 1PR8; GPMT; OZ0C*; 3NCY; AMMC; WR0O; 5Z3W; D2ZX; WRZB; 4RNL; CWYJ; SLWD; TLU2; BJ5W; 12CE; 8E6O*; VQB3*; RHQZ
textural feature (GLCM)	Joint Maximum*; Joint Average; Joint Variance; Joint EntropyLog2; Difference Average*; Difference Variance; Difference Entropy; Sum Average; Sum Variance; Sum Entropy; Angular Second Moment; Contrast; Dissimilarity; Inverse Difference; Normalised Inverse Difference; Inverse Difference Moment*; Normalised Inverse Difference Moment; Inverse Variance; Correlation; Autocorrelation; Cluster Tendency*; Cluster Shade; Cluster Prominence*	GYBY*; 60VM; UR99; TU9B; TF7R*; D3YU; NTRS; ZGXS; OEEB; P6QZ; 8ZQL; ACUI; 8S9J; IB1Z; NDRX; WF0Z*; 1QCO; E8JP; NI2N; QWB0; DG8W*; 7NFM; AE86*
textural feature (GLRLM)	Short Runs Emphasis; Long Runs Emphasis; Low Grey Level Run Emphasis; High Grey Level Run Emphasis; Short Run Low Grey Level Emphasis; Short Run High Grey Level Emphasis; Long Run Low Grey Level Emphasis; Long Run High Grey Level Emphasis; Grey Level Non Uniformity; Run Length Non Uniformity; Run Percentage	22OV; W4KF; V3SW; G3QZ; HTZT; GD3A; IVPO; 3KUM; R5YN; W92Y; 9ZK5
textural feature (NGTDM)	Coarseness; Contrast; Busyness; Complexity; Strength	QCDE; 65HE; NQ30; HDEZ; 1X9X;
textural feature (GLSZM)	Small Zone Emphasis; Large Zone Emphasis; Low Gray Level Zone Emphasis; High Gray Level Zone Emphasis; Small Zone Low Grey Level Emphasis; Small Zone High Grey Level Emphasis; Large Zone Low Grey Level Emphasis; Large Zone High Grey Level Emphasis; Grey Level Non Uniformity; Normalised Grey Level Non Uniformity; Zone Size Non Uniformity; Normalised Zone Size Non Uniformity; Zone Percentage; Grey Level Variance; Zone Size Variance; Zone Size Entropy	5QRC; 48P8; XMSY; 5GN9; 5RAI; HW1V; YH51; J17V; JNSA; Y1RO; 4JP3; VB3A; P30P; BYLV; 3NSA; GU8N

IBSI, Image Biomarker standardization Initiative.

**Table 3:** Optimal cut-off thresholds of SUVmax, MTV, TLG and RadScore area under the curve (AUC) of mid-term outcome, progression-free survival and overall survival in the training and validation cohorts.

Variables	AUC (95%CI)	Optimal thresholds
Mid-term outcome		
SUVmax	0.626 (0.523–0.728)	19.2
MTV	0.604 (0.503–0.705)	25.6
TLG	0.601 (0.500–0.702)	222.0
RadScore	0.753 (0.666–0.839)	2.0
Progression-free survival		
RadScore	0.620 (0.518–0.721)	2.2
Overall survival		
RadScore	0.627 (0.521–0.734)	2.2

CI, confidence interval; SUVmax, maximum standardized uptake value; MTV, metabolic tumor volume; TLG, total lesion glycolysis; RadScore, Radiomics Score.

### Univariate and multivariate analysis results

**Table 4** shows the between-group differences in clinical characteristics, PET metabolic indices regarding mid-term efficacy.

**Table 4:** Univariate and multivariate analyses of factors predictive of mid-term treatment outcome in the training cohort.

Category	Variables	Univariate analysis		Multivariate analysis	
		OR (95%CI)	*P* value	OR (95%CI)	*P* value
Clinical predictors	Sex, F/M	2.356 (1.078–5.148)	0.032	2.760 (1.196–6.368)	0.017*
	Age, <60/≥60	0.807 (0.374–1.740)	0.584		
	LDH level, normal/elevated	2.130 (0.823–5.513)	0.119		
	B symptoms, no/yes	3.647 (1.703–7.813)	0.001	4.065 (1.837–8.955)	0.001*
	ECOG PS, 0–1/≥2	0.667 (0.193–2.299)	0.521		
	IPI, 0–2/≥3	1.148 (0.441–2.985)	0.778		
	NCCN-IPI, 4–5/6–8	1.467 (0.524–4.109)	0.466		
	Ann Abor stage, I-II/III-IV	2.368 (0.471–11.909)	0.296		
	Extranodal involvement, 0–1/≥2	0.515 (0.174–1.526)	0.231		
	Bone marrow involvement, no/yes	0.787 (0.305–2.029)	0.620		
	Bulky disease, no/yes	0.853 (0.268–2.717)	0.788		
	Pathological type, GCB/non-GCB	0.982 (0.426–2.265)	0.967		
PET predictors	SUVmax	2.672 (1261–5.659)	0.010	2.619 (1.107–6.194)	0.028*
	MTV	3.108 (1.224–7.893)	0.017	2.331 (0.432–12.586)	0.325
	TLG	2.503 (1.109–5.649)	0.027	0.698 (0.155–3.156)	0.641
Radiomics predictors	RadScore	7.931 (3.257–19.314)	0.001	7.167 (2.815–18.248)	0.001*

CI, confidence interval; OR, odds ratio; F, female; M, male; LDH, lactate dehydrogenase; ECOG PS, Eastern Cooperative Oncology Group performance status; IPI, International Prognostic Index; NCCN-IPI, National Comprehensive Cancer Network International Prognostic Index; GCB, germinal centre B cell; SUVmax, maximum standardized uptake value; MTV, metabolic tumor volume; TLG, total lesion glycolysis; RadScore, Radiomics Score.

*P < 0.05.

For the clinical variables, we found that gender (OR=2.760 (95%CI:1.196–6.368), *P*=0.017) and B symptoms (OR=4.065 (95%CI:1.837–8.955), *P*=0.001) were independent risk factors for mid-term outcomes, as shown in **Table 4**.

Regarding the PET variables, RadScore (OR=7.167(95%CI:2.815–18.248), *P*=0.001) and SUVmax (OR=2.619 (95%CI:1.107–6.194), *P*=0.028) were independent risk factors influencing mid-term outcomes. These results were presented in **Table 4**.

### Assessment and validation of models built for predicting mid-term efficacy

**Figure 4:**
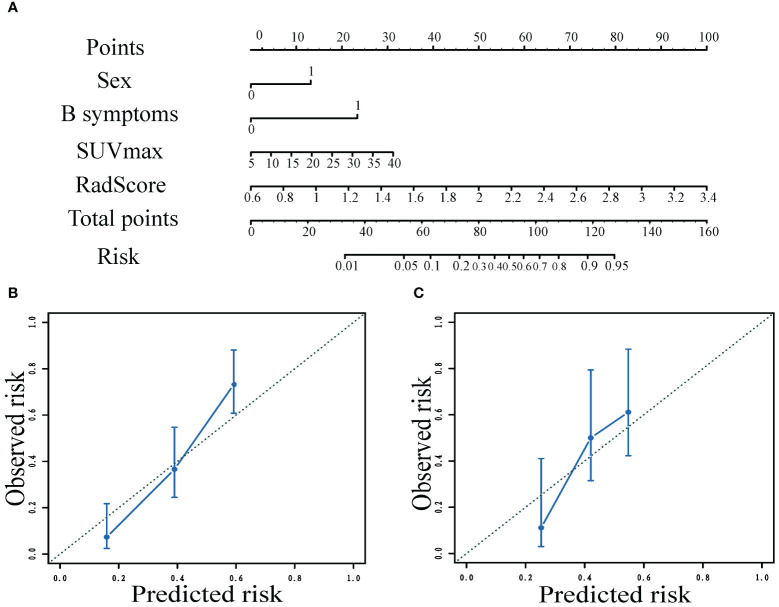
Nomogram to predict the patient mid-term efficacy risk **(A)**. Calibration curves of the model for predicting mid-term response in the training **(B)** and validation **(C)** cohorts.

To predict mid-term efficacy, we developed a combined model that utilized separate clinical predictors (gender, B-symptoms), PET predictor (SUVmax) and RadScore (**Figure 4**; **Table 5**). Additionally, we also created separate clinical models, PET-based models, IPI model and NCCN-IPI models (**Table 5**).

**Table 5:** The mid-term treatment outcome prediction models included in this study.

Models	Included variables
Combined Model	Sex; B symptoms; SUVmax; RadScore;
Clinical model	Sex; B symptoms;
PET based model	SUVmax;
NCCN-IPI IPI	NCCN-IPI IPI

IPI, International Prognostic Index; NCCN-IPI, National Comprehensive Cancer Network International Prognostic Index; SUVmax, maximum standardized uptake value; RadScore, Radiomics Score.

Nomograms visualized the score of risk factors on mid-term efficacy. The calibration curves after 1000 repetitions of bootstrapping for each model, which showed satisfactory agreement between the estimated values and the actual observed values in both the training and validation groups for the combined model (**Figure 4**).

**Figure 5:**
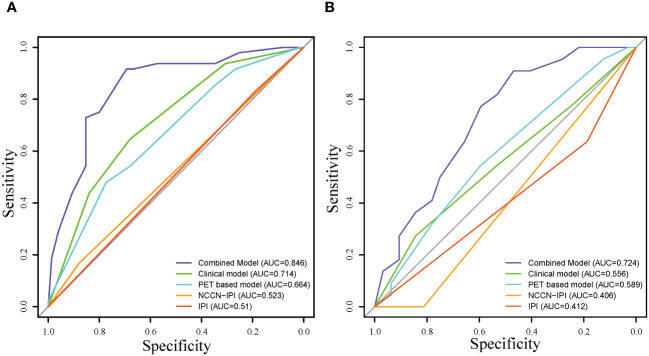
Receiver operating characteristic curve of the models for predicting mid-term response in the training **(A)** and validation **(B)** cohorts.

The ROC curves of the models for predicting mid-term response in the training (A) and validation (B) cohorts, which showed that the AUC values of the combined model (0.846;0.724)of clinical factors, pet metabolic parameters and RadScore were better than those of the clinical model (0.714;0.556), PET based model (0.664; 0.589), NCCN-IPI model (0.523;0.406) and IPI model (0.510;0.412) (**Figure 5**).

### Performance analysis of the combined models in clinical use

**Figure 6:**
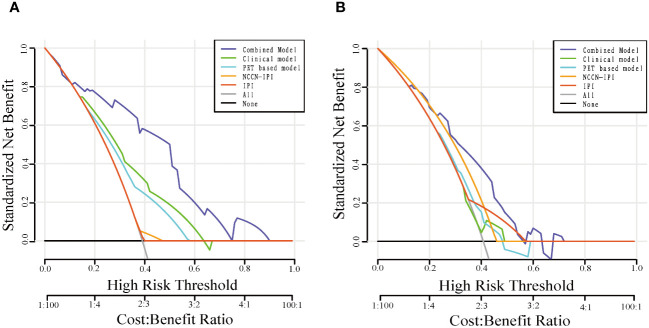
Decision curve analysis for the models in the training **(A)** and validation **(B)** cohorts.

DCA were shown in **Figure 6**. These analyses demonstrated that the combined model consistently outperformed the clinical model, PET-based model, IPI model and NCCN-IPI model in terms of overall net benefit for most risk thresholds in both the training and validation cohorts.

### Survival analysis in the training and validation cohorts

**Figure 7:**
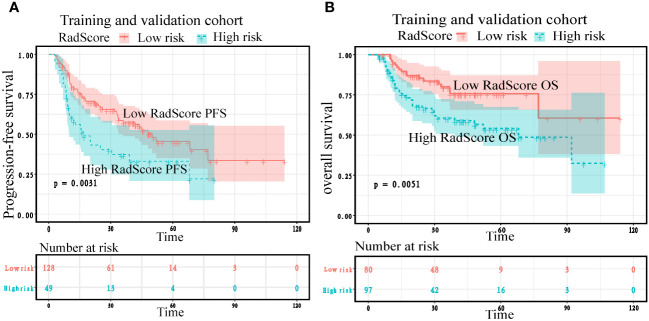
Kaplan–Meier plots according to RadScore for patients’ progression-free survival and overall survival in the training **(A)** and validation cohorts **(B)**.

To confirm the added prognostic value of RadScore, we evaluated it in low RadScore groups and high RadScore groups. The low and high-risk groups identified using the RadScore cut-off threshold demonstrated distinct outcomes in terms of PFS and OS in both the training and validation cohorts (**Figure 7**; **Table 6**). The prognosis power of the low RadScore group was superior to that of the high RadScore group.

**Table 6:** Cox regression of RadScore predictive of progression-free survival and overall survival in the training and validation cohorts.

	RadScore	Number	HR	HR (95% CI)	*P* value*
Progression-free survival	High/Low	49/128	2.1737	1.2983–3.6392	0.003
overall survival	High/Low	97/80	2.1356	1.2561–3.6309	0.005

HR, Hazard ratios; CI, confidence interval; RadScore, Radiomics Score.

^*^P < 0.05.

Cox regression analysis showed that the risk of adverse prognostic events in the group with high RadScore was higher than that in the group with low RadScore. Patients with a low RadScore (n=128) had a better PFS (Median months,50;95%CI:33.533,77.000) than those with a high RadScore (n=49) (Median months,17;95%:9.567,39.000). The risk ratio and 95% confidence interval of high RadScore group (n=49)/low RadScore group (n=128) were 2.1737 (1.298–3.639), and the *P* value were 0.003. Patients with a low RadScore (n=80) had a better OS (Mean months, 85.965;95%CI:72.684,99.246) than those with a high RadScore (n=97) (Mean months, 62.365;95%CI: 51.938,72.792). The risk ratio and 95% confidence interval of the high RadScore group (n=97)/low RadScore group (n=80) were 2.1356 (1.256–3.6309), and the *P* value were 0.005 (**Table 6**).

Kaplan Meier analysis showed that both in the training cohort and the validation cohort, the low RadScore group and the high RadScore group showed the same results in PFS and OS. However, the probability of adverse prognosis risk events in the low RadScore group was lower than that in the high RadScore group (**Figure 7**).

## Discussion

In this retrospective study utilizing real-world data, we found that a combined model, which incorporated RadScore, outperformed clinical, PET, and NCCN-IPI models in predicting mid-term efficacy and prognsis of DLBCL patients. This combined model can serve as a valuable tool for individualized outcome prediction and guiding treatment decisions for early-stage, high-risk DLBCL patients.

Accurately predicting the mid-term outcomes of DLBCL patients is crucial for optimizing treatment strategies. Numerous studies have endeavored to evaluate the predictive value of PET radiomics features for DLBCL. Santiago et al (35) demonstrated a models based on radiomics accurately predicted refractory DLBCL. Their study employed RF as a classifier, randomly assigning patients to training (70%) and independent test cohorts (30%). The AUC of the two cohorts was 0.83 and 0.79, respectively. Coskun et el. found that texture features extracted from baseline PET predicted chemotherapy insensitivity to R-CHOP regimens in DLBCL patients with an ROC accuracy of 0.87 (AUC=0.81). Notably, SUVmax and the differences in grey-scale covariance matrix played crucial roles in predicting chemotherapy insensitivity (36). Consistent with prior studies, our study independently associated the RadScore based on PET radiomic features with mid-term outcomes in high-risk DLBCL patients (OR=7.167 (95%CI:2.815–18.248), *P*=0.001). The RadScore on 11 key radiomic features obtained from PET were valuable in predicting the mid-term efficacy of high-risk DLBCL patients. This is likely attributed to the close association between radiomics features and tumor heterogeneity (37, 38), which serves as a prognostic determinant of patient survival (39, 40).

With the increasing utilization of machine learning techniques in extracting and classifying image features. The LASSO model is a selection method that effectively narrows down and regresses from a large pool of potentially multicollinear variables to obtain a set of relevant predictors (32). Many studies have employed the LASSO to identify and classify data features. However, existing studies often employ a single machine learning method for radiomic features selection and construction. In clinical practice, a machine learning method that combines feature classification and cross-validation can enhance the accuracy and generalization of the predicted results (41). In this study, we developed the cross-combination pairs of seven machine learning method generate 49 permutations, and determine the optimal feature selection-classification pairs based on the maximum AUC results to obtain the final RadScore. Our research method made RadScore more robust and reproducible than those studies with single machine learning method. **Figure 3** illustrated that the ET-LASSO model had a poor AUC (0.370), while AUC of the LASSO-LASSO model for predicting mid-term efficacy were 0.74. Additionally, our study revealed that the best LASSO-LASSO models selected radiomic features from shape feature (Surface Area), first-order features (Global Intensity Peak etc.) and texture features (GLCM), which indicated that main first-order features and texture features possess good ability to discriminate high-risk patients.

[18F]-FDG PET/CT can provide information about tumor biology by measuring cellular glucose metabolism. Our study demonstrated that SUVmax as an independent predictor of medium-term efficacy (OR=2.619 (95% CI: 1.107–6.194), *P*=0.028). The result were consistent with previous studies (42). We developed a user-friendly model that integrated RadScore, PET metabolic factors, and clinical risk factors and compared it with other models (e. g. clinical models, PET-based models, IPI model and NCCN-IPI model). The ROC curves and DCA results demonstrated that the combined model outperformed the other models, the performance of IPI model and NCCN-IPI model in the training cohort and the validation cohort were both unsatisfactory. Additionally, the combined model exhibited good agreement with the calibration curve and demonstrated a clear advantage in terms of AUC. These results indicate that the combined model is more suitable and practical for predicting medium-term outcome of DLBCL. Consistent with previous studies (43, 44), our results suggest that the IPI and NCCN-IPI may require improvement in identifying intermediate-high/high-risk DLBCL patients who would benefit from non-first-line treatment. Furthermore, our results support the RadScore of radiomic features(shape, first-order and GLCM) with SUVmax and clinical predictors, aligning with the findings of Jiang et al. (24, 45), to accurately identify intermediate-high/high-risk DLBCL patients.

One limitation of our study was its retrospective. We collected patient data from two medical centers, but future studies should include data from additional centers to ensure clinical generalizability. Given the specificity of DLBCL, the distribution of intra- and/or extra-lymph node lesions are highly variable and heterogeneous. The morphological and textural features of the lesions are highly sensitive to tumor segmentation methods. Thus, we employed the 41% of SUVmax tumor segmentation method recommended by the European Association of Nuclear Medicine. This method may be more practical and straightforward to implement in clinical. Additionally, the use of mid-term PET as the study endpoint in our research may lead to false-positive interpretation results. To justify therapeutic decisions, complementary studies utilizing end-stage PET should be conducted in the future. However, a major strength of our study lies in the homogeneity of the included patients, as they all had new-onset DLBCL histology and received R-CHOP-like regimens as standard treatment. The methodology employed also supports the general applicability of our model.

## Conclusion

The RadScore is obtained by the feature selection-classification crossover combination of 7×7 machine learning method that included shape feature, first-order features and texture features (GLCM), can serve as a predictor for both mid-term efficacy and prognosis in DLBCL patients. In addition, the combined model which integrates the RadScore, PET metabolic indicator (SUVmax), and clinical risk factors (sex, B symptoms), can aid in rational risk stratification and facilitate the screening of appropriate treatment regimens for at intermediate-high/high risk DLBCL patients in the early stages.

## Data availability statement

The raw data supporting the conclusions of this article will be made available by the authors, without undue reservation.

## Ethics statement

The studies involving humans were approved by Ethics Committee of Nanjing Drum Tower Hospital of Nanjing University Medical School and West China Hospital of Sichuan University. The studies were conducted in accordance with the local legislation and institutional requirements. The participants provided their written informed consent to participate in this study. Written informed consent was obtained from the individual(s) for the publication of any potentially identifiable images or data included in this article.

## Author contributions

MC: Investigation, Visualization, Writing – original draft. JR: Methodology, Writing – review & editing. JZ: Formal Analysis, Supervision, Writing – review & editing. YT: Validation, Writing – review & editing. CJ: Conceptualization, Writing – review & editing. JC: Software, Writing – review & editing. JX: Funding acquisition, Writing – review & editing.
